# From active treatment to surveillance: How the barriers and facilitators of implementing survivorship care planning could be an opportunity for telehealth in oncology care for rural patients

**DOI:** 10.21203/rs.3.rs-3117303/v1

**Published:** 2023-06-30

**Authors:** Jenn Alford-Teaster, Danielle Danielle Vaclavik, Inger Imset, Jenna Schiffelbein, Kathleen Lyons, Nirav Kapadia, Ardis Olson, Elizabeth McGrath, Karen Schifferdecker, Tracy Onega

**Affiliations:** Dartmouth College; Dartmouth College; Dartmouth–Hitchcock Medical Center; Dartmouth College; MGH Institute of Health Professions; Dartmouth–Hitchcock Medical Center; Dartmouth–Hitchcock Medical Center; Dartmouth–Hitchcock Medical Center; Dartmouth College; Huntsman Cancer Institute

**Keywords:** survivorship, telehealth, tele-oncology

## Abstract

**Purpose:**

Cancer survivorship care planning is a recognized yet underutilized aspect of care delivery and the opportunity for telehealth in cancer survivorship is examined.

**Methods:**

We conducted a mixed-methods study in Vermont and New Hampshire to characterize perceptions of rural cancer providers and survivors regarding survivorship transitions in care, consisting of: a) key informant interviews with primary care and oncology clinicians, b) a broader survey of clinicians, and c) surveys and focus group discussions with cancer survivors. In these interactions, we also explored the use of a shared telehealth survivorship care planning appointment between oncology clinicians, primary care clinicians, and survivors

**Results:**

Results from surveys and interviews clustered around several themes, namely: 1) infrequent care transitioning back to primary care; 2) lack of mental health services; 3) lack of side effect education; 4) low perceived utility of survivorship care plans; 5) clinicians exclusively communicate using the EMR and finding it imperfect; and 6) clinicians and survivors reported conflicting perceptions regarding survivors’ access to telehealth options.

**Conclusions:**

Our results suggest that telehealth has potential to augment the delivery of survivorship care planning; however, key technical and logistical concerns need to be addressed, particularly enhanced coordination across clinician scheduling and ensuring payment parity for various telehealth implementation strategies.

**Implications for Cancer Survivors:**

Cancer survivorship care planning is a recognized yet underutilized aspect of care delivery. There is an opportunity for the application of telehealth for supportive care in survivorship care planning, which should be a focus of further research.

## Background

Survivorship care planning refers to an orderly process by which clinicians and survivors navigate the transition from active treatment to post-treatment.^1–3^ A “cancer survivor” is defined by the National Cancer Institute (NCI) as any individual with a history of cancer beginning at the time of diagnosis, through treatment and surveillance.^4^ The importance of survivorship care planning has been underscored recently by the American College of Surgeons’ Commission on Cancer (Standard 4.8), which details elements to promote and enhance the use of survivorship care plans and includes goals for the development of survivorship care programs within hospitals^5^. However, implementing survivorship care plans is complex and can be curtailed due to limited understanding of how best to implement the plans themselves, as well as imperfect, asynchronous communication among multiple clinicians and their patients.^6,7^ Therefore, methods for improving communication among clinicians and patients are needed.

Telehealth is one vehicle for addressing these gaps^8–10^. While it has the potential to reach underserved rural and remote cancer patient populations, telehealth utilization in cancer care has been limited due to concerns regarding poor technology infrastructure and insufficient understanding of best practices for implementation throughout the cancer care continuum.^8,11–15^ With the novel coronavirus (COVID-19) pandemic came an opportunity to evaluate telehealth in several care settings., including cancer survivorship care ^8,9,16,17^. In previous work, we demonstrated clinicians’ interest increased along with an increase in understanding of applications of telehealth, including its potential utilization in cancer care^8^. Specifically, telehealth holds potential for the delivery of supportive cancer care, such as telepsychiatry or nutrition counseling^8^. However, there are communication and coordination gaps during the transition from active cancer treatment to surveillance.

In this study, we sought to understand survivor and clinician perceptions regarding survivorship care planning and explored the possible role for telehealth to improve these interactions.^8–10^.

## Methods

To identify actionable themes related to the transition from active cancer treatment to surveillance, a study team at Dartmouth Cancer Center (DCC) conducted a multi-method study consisting of: a) key informant interviews with primary care and oncology clinicians b) a survey of a broader cohort of oncology and primary care clinicians, and c) a survey and focus group discussions with cancer survivors. Study procedures and materials were reviewed and approved by the Dartmouth-Hitchcock Health Institutional Review Board (IRB). The methodological approach was tiered in order to elucidate insights by first interviewing participants (e.g. providers and survivors separately), and then following up with additional survey work to enhance understanding of barriers and facilitators.

## Setting

Given that 80.7% of DCC patients reside in rural areas^18^, we were interested in specifically exploring barriers and facilitators of rural survivors’ transition from active cancer treatment to surveillance. As such, the study was carried out in the Dartmouth Cancer Center (DCC) catchment area which includes the rural setting of Vermont (VT) and New Hampshire (NH).

## Key Informant Interviews with Clinicians

We conducted key informant interviews with primary care and oncology clinicians via Zoom^™^ in late summer 2020. Prospective interviewees were recruited initially through email invitations, then through “snowball” recruitment by asking interviewees to recommend and make connections to other prospective clinicians to interview. We collected information about the clinicians’ roles and practice locations, as well as information about their cancer patients, including the approximate percentage living in rural areas, via a brief survey prior to the interview. All interviewees consented to participate in the study and were offered a $50 Amazon.com gift card for their participation.

Interviews were led by a trained moderator who used a semi-structured interview guide developed by the study team. The guide was designed to elucidate interviewees’ perceptions of survivorship logistics and care plans, communication practices and preferences, and a hypothetical scenario using telehealth. In this hypothetical scenario, we asked interviewees for their feedback regarding a shared telehealth survivorship care planning appointment involving their patient, the treating oncology clinician, and the patient’s primary care clinician, during which they would review survivorship care planning and facilitate the transition out of active cancer care.

Interviews lasted 30–40 minutes and were recorded and transcribed. See Appendices A and B for a full list of interview questions.

## Survey of Primary Care and Oncology Clinicians

We also conducted surveys with primary care and oncology clinicians within NH and VT. Electronic invitations were sent to clinicians from two academic medical centers in NH and VT, as well as a network of regional primary care clinicians interested in research opportunities. Prospective participants accessed our online survey via Qualtrics^™^, where they consented to participate and completed eligibility questions. To be eligible for the survey, prospective participants needed to practice primarily in New Hampshire or Vermont and be a Medical Doctor (MD), Doctor of Osteopathic Medicine (DO), Advanced Practice Registered Nurse (APRN), Physician Assistant (PA), or Nurse Practitioner (NP). Primary care clinicians also needed to work in Internal Medicine, Family Medicine, or General Practice settings and practice in a rural community, as defined by classes 7—9 of the Rural-Urban Continuum Codes^19^. Oncology clinicians needed to work in Medical, Hematological, Radiation, Surgical, or Gynecological Oncology settings. Those eligible were then able to complete our survey. Participants who completed the survey were entered into prize drawings to receive a $100 Amazon.com gift card.

Surveys were specific to clinicians’ specialty—primary care or oncology—but were complementary and designed to explore themes unique to their work. Themes included: a) their actual and perceived roles and responsibilities during survivors’ transition out of active cancer treatment, b) challenges encountered during the care transition and ways to improve the transition, c) familiarity with and use of survivorship care planning, and d) telehealth’s utility in supporting the care transition—including whether the clinicians already used or would use telehealth to facilitate a shared appointment with their patient, themselves, and another care team. See Appendices C and D for survey questions.

## Survey and Focus Group Discussions with Survivors

Finally, we conducted a survey and focus group discussions with cancer survivors from rural NH and VT. Prospective participants were invited to participate via social media and through partner organizations (e.g., Vermonters Taking Action Against Cancer, NH Breast Cancer Coalition) sharing the study information with their networks. Prospective participants accessed an online survey via Qualtrics^™^, where they consented to be in the study and completed eligibility questions. To be eligible, participants needed to live in a RUCC 7—9 county of NH or VT; have been diagnosed with stage 1, 2, or 3 cancer as an adult; have received chemotherapy, endocrine therapy, immunotherapy, or radiation therapy; have completed their cancer treatment in the past 10 years; and be receiving care from a clinician other than their oncology clinician. Eligible participants were then able to complete our survey, which included questions about their demographics, cancer care experience, their experience during the transition out of active cancer care, perception of survivorship care planning, access to different technologies, use of technology to conduct health-related activities, and contact information (see Appendix D for survey questions). Participants who completed the survey and provided valid contact information received a $10 gift credit for Amazon.com and were later invited to participate in our focus group discussions.

We hosted two asynchronous, online focus groups. Each ‘bulletin board’ style focus group was conducted over a three-week period in Fall/Winter 2020 using FocusgroupIt.com^™^. Participants earned $10 gift credit for Amazon.com for each week they participated. Each week, participants were asked to respond to a set of core questions and probes from the focus group moderator and to interact with other participants. The core questions focused on exploring survivors’ cancer care experience—both during active treatment and during the transition out of active treatment. They were also asked to discuss challenges they and their families experienced, what supports and resources they received or wish they had received, how they perceived communication among clinicians and survivors, their experiences with telemedicine, and their suggestions to improve future care. As we did with clinicians during the key informant interviews, we also asked survivors for their feedback on the concept of a shared survivorship care planning appointment with their primary care and oncology clinicians, facilitated by telehealth. See Appendix E for focus group moderator guide.

## Analysis

Survey data were summarized in Stata SE for Windows^©^. Interview and focus group transcripts were imported into Dedoose^™^, a qualitative analysis software program and thematically coded by professional qualitative analysts. Focus groups were conducted by authors JS, II. Key Informant Interviews (KII) were conducted by DV, KAS, and JAAT. Two researchers, DV and KES, developed codebooks and analyzed the data for themes across participant groups to prepare summaries on themes present in the work. Themes were triangulated by researchers/authors DV and KES on topics that overlapped across clinician interviews and survivor focus groups.

## Results

The clinician key informant interviews (n = 15) included responses from four oncology clinicians, four primary care clinicians, two oncology Advance Practice Registered Nurse (APRN)s, one primary care APRN, two oncology Nurse practitioners (NP), one PCP/NP, and one radiation oncologist. The clinician surveys included 11 primary care clinicians and 40 oncology clinicians; however, 3 of the 40 oncology clinicians’ surveys were incomplete and, therefore, not analyzed. The survivor survey was completed by 25 survivors, and 16 of them also participated in the focus group discussions. See Table 1 for a summary of study participants and Appendix G for study participant demographics by method (Focus Group, Key Informant Interview, and Surveys).

When asked what forms of telehealth they currently used, both primary care clinicians (n = 6, 55%) and oncology clinicians (n = 27, 73%) reported web-based patient portals as the most used tool. Additionally, clinicians in the survey reported interest in a shared telehealth appointment involving clinicians, patients, and other care team members (91% of primary care clinicians (n = 10); 32% of oncology clinicians (n = 10)). However, 55% (n = 6) and 32% (n = 10) primary care clinicians and oncology clinicians, respectively, reported billing and payment considerations limiting their use of telehealth.

Among survivors who responded to the survey questions, 100% (n = 23) reported having internet access at home and 83% (n = 19) reported using the internet to communicate with a clinician in the past 12 months. When asked whether they recalled receiving a survivorship care plan when they completed cancer treatment, 67% (n = 16) reported not having received a survivorship care plan; among those that recalled receiving a survivorship care plan (33%, n = 8), only one respondent could confirm that their ‘main doctor’ had received a copy.

Through our qualitative analysis of the clinician interviews and survivor focus groups, we identified the following seven themes: 1) Survivors are not transitioning back to primary care. 2) Survivors need more access to mental health services. 3) Survivors want more education about side effects. 4) Survivorship care plans sound good in theory but primary care clinicians may not get them, and research on effectiveness is inconclusive. 5) Clinicians almost exclusively communicate using the EMR, and it is an imperfect system. 6) Clinicians and survivors gave conflicting reports on survivors’ access to telehealth options. 7) Clinicians and survivors liked the shared telehealth survivorship care planning appointment concept, but clinicians felt it was unrealistic.

### Survivors are not transitioning back to primary care.

An assumption going into the project was that survivors transition mostly or completely away from their oncology team back to primary care during survivorship. This assumption was not supported by either the clinician interviews or the survivor focus groups.

“This is another elephant in the room…patients often don’t want to go back to their primary care physicians about this. Nothing against their primary care, but they often feel like they’re being let go or let down, and they feel that they finally have a strong, trusting relationship, and that you’re just sort of handing them off” (oncology clinician).

“The thing that happens most often nowadays is their [Primary Care Clinician (PCP)] has left and it’s really difficult to get into a new PCP. Or they met their new PCP, but they don’t like them. So, what happens is our patients get very attached to us because we spend so much time with them. We get them at that really vulnerable point. We develop really strong personal relationships… I think that’s probably our biggest challenge with this is that our patients are not plugged in nor do they feel like they have that relationship with the PCP like they do with us” (oncology clinician).

### Survivors need more access to mental health services.

Survivors who struggled during survivorship reported depression, anxiety, and PTSD. While oncology clinician interviewees reported that they often refer survivors to mental health services, survivors may not be receiving enough mental health support. More exploration of this disconnect is needed.

“I didn’t have any “instructions“ on how to live my life as a survivor…It would be nice to have a ‘holistic’ treatment plan for survivorship. Something that includes diet, exercise, medication, stress, supplements, things to avoid, screenings/tests, appointments” (patient).

### Survivors want more education about side effects.

The most common refrain from survivors while discussing active treatment and survivorship was how surprised they were by side effects. Both survivors and oncology clinicians discussed the oncology team going over side effects, yet survivors still felt like they were not educated enough about how their medications would affect them both in the short and long term. Further exploration of how and when this information should be given is needed to make sure survivors receive the information.

“I knew about monitoring/scan timelines, but I didn’t know about lymphedema, anxiety/depression, etc. These “other” side effects of cancer should be part of the conversation, too” (survivor).

### Survivorship care plans sound good in theory but primary care clinicians may not get them, and research on effectiveness is inconclusive.

Oncology clinicians, primary care clinicians, and survivors like the idea of survivorship care plans. However, primary care clinicians may not be receiving them, and oncology clinicians who are responsible for doing the care plans are unclear on their usefulness or effectiveness.

“I believe a copy is in my medical records for my main MD to see. I am glad that I have it. I have not used it since it was written but I have it with my important papers in case I need to share it with someone/another doctor” (survivor).

“I don’t know that I’ve ever received a survivorship plan, but honestly, the hard truth is that when there are pages and pages and pages in the document, you might try to find the one thing that makes sense to you that summarizes it. You may not go through – sometimes we’ll get patient instructions that include things like what to do about a port infection or something like that on a patient doesn’t even have a port. So, there’s a lot of information that comes to us that is not relevant. And so maybe there’s something really relevant in there that I’ve missed, it’s very possible” (PCP).

### Clinicians almost exclusively communicate using the EMR, and it is an imperfect system.

Clinicians reported frequent communication issues caused by the EMR that could potentially influence patient care. All clinicians felt there was much room for improvement in their EMR software but were making do with what they currently use.

### Clinicians and survivors offered conflicting reports on survivors’ access to telehealth options.

Clinicians reported survivors have issues with communicating outside of office used some form of telehealth in the past and found it to be a good way to augment care management, but not to replace in-person visits because of a lack of technology skills and/or a lack of access to technology. Inversely, survivors in the focus groups were comfortable using telehealth and patient portal options to communicate with their clinicians. Further exploration of survivor communication preferences and access is needed.

### Clinicians and survivors viewed a shared telehealth survivorship care planning appointment favorably, but clinicians felt it was unrealistic.

When prompted with the hypothetical telehealth intervention, participants generally agreed that telehealth offers the opportunity for strengthening transition care by enhancing clinician relationships with patients and limiting drive time to appointments but is dependent on available technology for both patients and clinicians, scheduling availability for the clinicians, and the necessary approvals for insurance coverage for telehealth. While they liked the idea, clinicians also felt the shared telehealth survivorship care planning appointment was unrealistic in their current care model. Perceived benefits and barriers are listed in Table 2.

If the shared telehealth survivorship care planning appointment were to be instituted in any regularity, systemic barriers—such as improved infrastructure and logistics—would need to be addressed in order to make this option feasible. When asked how long this appointment would need to be, clinicians’ answers ranged from 5 to 30 minutes, with most in the 15–20-minute range. Several oncology clinicians stated that primary care clinicians did not need to be present for the entire appointment (e.g., 5–10 minutes of a 60-minute survivorship appointment). Both primary care and oncology clinicians suggested having the appointment take place in a clinician’s office to reduce technology access and skill disparities among survivors. Survivors pictured doing the appointment from their home or from the oncology office.

“It is like a school parent-teacher conference, everyone gets filled in on what happened, what to look for, what may still happen and how to navigate post cancer” (survivor).

“I think it is a great idea. I think it is totally unrealistic” (oncology clinician).

## Discussion

The purpose of this study was to identify the barriers and facilitators associated with transition from active cancer treatment to survivorship, with a particular focus on exploring the feasibility and role of telehealth in potentially supportive interventions for survivorship care. Transitioning out of active cancer treatment is an ongoing process that involves careful coordination between oncology care teams and primary care clinicians. It often unfolds in an asynchronous way, unintentionally creating communication barriers between coordinating care teams and survivors. Due to the asynchronicity, unique survivorship care needs that do not fall within the explicit domain of an oncology clinician or a primary care clinician may go unaddressed. Specifically, survivors in our study reported unmet clinical and care needs. Telehealth delivery of specific services were not discussed as an alternative to in-person care, yet participants noted that telehealth intervention has the potential to meet these needs as long as certain criteria are met; specifically, availability of technology, scheduling, and insurance coverage^8,9^. Telehealth as an additional care coordination tool has potential because it does not require clinicians or survivors to travel long distance for their care and can be flexible in the delivery of care (e.g. video, telephone, and/or both), depending on the service needed.

It is commonly thought that survivorship care planning is desirable for survivors and somewhat so for clinicians but is not well-operationalized due to time/staffing, conflicting evidence on benefits, and missing components that would be helpful for survivors^20–23^. The survivors in our study report that they had used some form of telehealth in the past and found it to be a good way to augment care management, but not to replace in-person visits. Specifically, study participants supportive of the shared telehealth survivorship care planning appointment, though such a visit was thought to be infeasible from clinician perspectives. The pandemic has shown that some of these barriers may be reduced by utilizing telehealth for specific aspects of the survivorship ^9,15,24^. For example, within our study, communication among clinicians was almost exclusively through the electronic health record (EHR), but survivors primarily communicated during in-person visits. The pandemic has provided a necessary window into the benefits of telehealth in a way not previously understood. And since our study was conducted in the year of the pandemic unfolding, the utility of telehealth in the application of implementing survivorship care planning was not fully understood.

Expanding on the survivor and clinician sentiment that telehealth could serve to augment, but not replace, in-person care, we can begin to explore potential opportunities for the appropriate application of telehealth services in the context of survivorship. For example, this could be addressed by providing direct access to telepsychiatry appointments as coordinated by the survivorship team. Telepsychiatry for cancer survivors is a promising intervention because the application of telehealth in mental health support allows survivors the opportunity to receive treatment in the comfort of their own home; and is well documented as an effective alternative to in person visits for this type of care^15,17^.

Moreover, another area for consideration is the delivery of supportive education via telehealth on symptom management as well as short- and long-term side effects post cancer treatment. The most common refrain from survivors in our study while discussing active treatment and survivorship was how surprised they were by the short- and long-term side effects of the cancer treatment. Both survivors and oncology clinicians discussed the oncology team going over side effects, yet survivors still felt like they weren’t educated enough about how their medications would affect them both in the short and long term. Telehealth can be used for symptom management with a specific intention of exploring potential short- and long-term side effects and explaining what is normal and how to reduce the impact without asking survivors to make the trip to the medical office.^25^. These types of telehealth benefits offer flexibility and have the potential to address the gaps in survivorship identified in this study.

A limitation of this study is that the project progressed during the height of the first wave of the COVID-19 pandemic, and, as such, the initial survey outreach included at least 100 more clinicians. This suggests that during the period of open recruitment in December 2020 – early January 2021, the COVID19 pandemic possibly reduced availability of clinicians due to the increased workload associated with COVID related care and as such the ability to complete our surveys and interviews were strained.

Additionally, one key healthcare organization experienced a cyber-attack which impacted outreach through their email system, so recruitment to clinicians in that health system were limited access to them during the study timeframe. However, our findings indicate that further evaluation of telehealth as it applies to survivorship care planning is warranted, particularly as telehealth’s general and sustained use has expanded during the pandemic.

## Conclusions

Our study suggests that there are opportunities for appropriately placed telehealth appointments in the context of survivorship. Specifically, potential candidates for telehealth services for augmented survivorship care transition from active treatment to surveillance could include telepsychiatry, nutritional counseling, ongoing financial support counseling, and transition from oncology to primary care. While telehealth holds potential for improving the implementation of survivorship care plans it should be noted that even with the interest in this modality participants do not want it to replace the in-person visits entirely because it may be difficult to find support for routinely implementing this intervention due to multiple logistical issues. As such, further study is warranted to understand the most appropriate timing for a telehealth intervention to support the transition to survivorship.

## Figures and Tables

**Table 1:** Summary of study participants.

	Sample size (n)	Description

**Key Informant Interviews with Clinicians**	15	Clinicians working in primary care and oncology, including MD/Dos, APRNs, and NPs

**Survey of Primary Care and Oncology Clinicians**	51	11 clinicians from primary care
		40 clinicians from oncology (3 incomplete and removed from analyses)

**Survey of Cancer Survivors**	16	25 surveys (1 incomplete and removed from analyses)
**Focus Group Discussions with Cancer Survivors**		10 in Focus Group 1
		6 in Focus Group 2

**Table 2:** Perceived benefits and barriers of the shared telehealth survivorship appointment.

Benefits	Barriers

• **Patient would like it** (seeing the clinicians talk and being able to ask both questions)	• **Scheduling** both clinicians for the same time block (in the office different days, different appointment lengths, one doctor running late)
• It would make sure **clinicians are on the same page** on who is handling which parts of treatment	• Many patients (and some offices) don’t have the necessary **technology** to make it happen
○ It would **build relationships** between clinicians	○ **Billing insurance** so both clinicians get paid without overcharging patients
• It would provide **good information** for the patient and primary care clinician	
